# Controlled anarchy

**DOI:** 10.1038/s44319-025-00468-8

**Published:** 2025-05-13

**Authors:** Fiona M Watt

**Affiliations:** Directors’ Unit, EMBL Heidelberg, Meyerhofstr. 1, 69117 Heidelberg, Germany

**Keywords:** Economics, Law & Politics, History & Philosophy of Science, Science Policy & Publishing

## Abstract

When it comes to asking for government funding, life scientists can come over as anarchic, self-interested and unable to achieve consensus. While physicists are good at mobilizing behind ‘moonshots’, life scientists tend to work best in the context of a grass-roots movement.

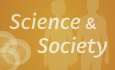

The existence of the pharmaceutical and biotech industries—along with all the products they have developed over decades—shows that investment in life-science research yields enormous benefits for human health, as well as agriculture and the environment. However, it is not always possible to predict what and when that investment will deliver. Whereas physicists often need to build consensus and large teams around clearly formulated research questions because of the sheer size of research projects in astronomy, high-energy or nuclear physics, some of the greatest discoveries in the life sciences were founded on serendipity. Penicillin, for example, was described by Alexander Fleming as “a mould which was not wanted” because it contaminated his bacterial culture plates. Even when biologists are able to define and rally around a clear goal—such as sequencing the human genome—it is not a given what the benefits will be.

Even when biologists are able to define and rally around a clear goal—such as sequencing the human genome—it is not a given what the outcome will be.

How then should governments make decisions about funding life-science research if the benefits are often not predictable? Should they ask for more clearly defined research questions and goals? Or should they simply provide a generous budget and leave it up to individual scientists to pursue their own research interests? Either approach will eventually spawn great discoveries, but the reality is that governments have many competing calls on their budget and need to have some understanding of what their investment could deliver. In addition, the boundary between basic research and application can be fuzzy, and the life sciences benefit disproportionately from philanthropic, pharma and biotech investment in addition to government funding.

Against this backdrop I convened an EMBO workshop to discuss how life scientists can be more effective at making the case for funding. We discussed the importance of providing trustworthy advice to governments and looked at how a country’s history and economic imperatives can drive different approaches to public and private investments. We also considered examples of ‘big science’ projects—their origins and ingredients for success—and whether these could be a model for funding mechanisms in the life sciences. Finally, although the participants spanned different continents, generations and expertise, we all agreed that funders face a particular challenge: discovery in the life sciences is essentially controlled anarchy rather than organised teamwork.

… discovery in the life sciences is essentially controlled anarchy rather than organised teamwork.

## History lessons

Governments need high-quality science advice and trusted sources of information, such as the EU Scientific Advice Mechanism or the US National Research Council, are available. However, it can sometimes be ignored. In my experience it can be frustrating when scientists are convened to offer advice but suspect that the outcome has already been decided—a tell-tale sign is when a bureaucrat offers to summarise the meeting with the chilling phrase ‘I’ll hold the pen on this’. This is why trusted individuals can be hugely important, both to the scientific community and to governments. The story of two, rather different, scientists illustrates this point. Virginijus Šikšnys of Vilnius University, well known for his research on the enzymes of nucleic acid metabolism, has played a major role in shaping research in Lithuania, a country in which, remarkably, the biotech sector accounts for approximately 2.5% of GDP. Nobel Laureate Sydney Brenner spent much of his career in the UK but subsequently worked in the USA, Japan and Singapore; his impact on research policy and investment was particularly strong in Singapore. Nonetheless, the problem for governments seeking expert advice is to understand who is trustworthy and to whom they should listen.

In terms of investment in the life sciences, history is important, as illustrated by Lithuania and Slovenia. In the 1970s, the USSR encouraged research and development of enzyme manufacture in Lithuania, while in Slovenia the focus of the Yugoslavian government was on developing a nuclear reactor. The Jožef Stefan Institute in Ljubljana, still the largest research institute in Slovenia, was founded in 1949 for nuclear research, although the institute’s role expanded to include molecular biology and biotechnology. After Lithuania broke away from the USSR in 1990 and Slovenia declared its independence in 1991, both countries have seen significant investment from pharma/biotech companies that are attracted by their highly educated workforce, with Lithuania an important base for Thermo-Fisher, and Novartis the first innovative medicines company to establish research and production facilities in Slovenia. Multi-national companies in need of skilled labour may influence governments in a way that universities cannot. Thus, investing in life sciences can become a win-win situation for industry and government—profit and products for the company and job creation and economic growth for the country.

History and politics are only two elements of the research ecosystem that determine whether life science research can flourish. A funding mechanism that enables individual scientists to conduct their own independent research is important, together with a diversity of public and private funding sources and merit-based review mechanisms. Favourable business conditions, industry maturity, infrastructure and manufacturing are all part of this innovation ecosystem. A sensitivity to the specific needs of biomedical research is also important: Venture Capital investors have long recognised that life-science companies often suffer scale-up challenges and take a longer time to become profitable. Singapore represents an unusual ecosystem as it benefits from Temasek, an investment company whose shareholder, the Minister of Finance, holds the management accountable for success without directing strategy.

## Big science

‘Big science’ projects are large-scale, government-funded research endeavours with a clearly stated objective; they involve large teams of researchers working together over several years and often require new technology development. The first big-science project was the Manhattan project to develop the atomic bomb. From World War II on, physicists have been in high demand to develop new weapons, and improve communication methods and energy production. To this day, many of the big projects in physics are directly related to defence and energy, and thus more readily gain government buy-in. In contrast, transformative research in biology tends to start small and then grow exponentially—for example, the discovery of penicillin kick-started the modern pharmaceutical industry while recombinant DNA technology founded the biotech industry.

… many of the big projects in physics are directly related to defence and energy, and thus more readily gain government buy-in.

Nevertheless, life scientists do have a taste for Big Science projects. The first of these, the Human Genome Project, was led by an international group of researchers co-funded by commercial, philanthropic and government sources. Despite initial scepticism that it was worth even attempting (Robertson, 1986), the successful sequencing of the human genome has not only benefitted human health but also provided new technologies and tools for researchers and clinicians. Still, compared to their colleagues in the physical sciences—many of whom also work in small individual teams on smaller projects—biologists have found it much harder to convince governments to support large-scale projects in the life sciences. Paradoxically, it may be easier to get popular support for research into something almost no-one understands, such as the Higgs Boson, than for something—combatting disease—that everyone agrees is a good thing. It is thus unfortunate that the ‘war on cancer’ under the US Nixon administration raised unrealistic expectations, because despite tremendous progress in cancer prevention, diagnosis and treatments, no-one can claim that cancer has been eradicated.

Paradoxically, it may be easier to get popular support for research into something almost no-one understands, such as the Higgs Boson, than for something—combatting disease—that everyone agrees is a good thing.

Two more recent Big Science projects in the life sciences are the Human Cell Atlas (Regev et al, 2017) and the Human Brain Project (Frégnac and Laurent, 2014). The Human Cell Atlas is a bottom-up initiative, launched in 2016 after two years of intense discussions and planning. It received an early boost from the Chan Zuckerberg Initiative and has subsequently gathered momentum with different sources of government and non-government funding from around the world. In contrast, the Human Brain Project (2013–2023) was an EU Flagship project with up-front funding of €1 billion. While the brain project undoubtedly funded valuable research, it suffered from problems with the leadership structure and internal disputes about priorities. It became, some would argue, a predatory alliance of scientists motivated by access to funding rather than achieving a common goal.

There clearly is a natural scale for taking on problems and it is not worth embarking on a big project unless there is a critical mass of scientists who agree that it should be attempted. In this regard, bottom-up initiatives, such as the Human Genome Project or the Human Cell Atlas, may work better than top-down directives. Life scientists want inspiring things to do—if they have sufficient funding for their own research, they are happy to be part of something bigger too.

## Pragmatism

While ‘Big Science’ projects have an aspirational flavour, some successful innovations in the life sciences are serendipitous or arise from pragmatic considerations. One example is the Francis Crick Institute, which is widely recognised as one of the world’s premier life science research institutes since it became operational in 2017. The ingredients for success included two older, pre-existing institutions, one with government and one with charitable funding, both of which were in outdated buildings in London. A further ingredient was that the UK government owned land originally ear-marked for the British Library, which had lain undeveloped for many years and thus provided an ideal location for the new Institute. Another, different example is the SARS-CoV-2 pandemic because it illustrates how a diverse biomedical community that was forced to work as a single entity in a time of crisis learned the benefits of long-term cooperation. In April 2020, the German Federal Ministry of Education and Research (BMBF) created the University Medicine Network to bring together and evaluate action plans, diagnostics and treatment strategies from all university hospitals to ensure the best possible care for COVID-19 patients throughout the country. The structures that were established for rapid and uniform collection and consolidation of clinical data were so successful that the Network has continued post-pandemic.

In making the case for better financial support of life-science research, the quality and honesty of the scientific community are important factors to gain governments’ attention and funding. However, given the anarchic nature of the life sciences, there may be a cacophony of different opinions rather than clear guidance and advice on how to invest efficiently in research. As history has shown, it is therefore important that a few trusted and influential individuals, known for the quality, honesty and generosity of their advice, can provide guidance. We hypothesise that, as a starting point, the community should convene a small number of respected scientists to come up with, say, 10 ideas for future research directions. Explaining those big ideas—rather than big projects—in a way that politicians and the population at large can understand and appreciate may help to secure future funding for the benefit of science and society.

In making the case for better financial support of life-science research, the quality and honesty of the scientific community are important factors to gain governments’ attention and funding.

## Supplementary information

Peer Review File
