# Supplementary material for: Controlled anarchy
Source: EMBO Rep. 2025 May 13;26(11):2800–2. doi: 10.1038/s44319-025-00468-8 (PMC12152184; doi:10.1038/s44319-025-00468-8)
Supplement: Supplementary file 1 — Peer Review File [file 44319_2025_468_MOESM1_ESM.pdf]

EMBO reports

Controlled anarchy: how life scientists can gain the trust of governments

Fiona Watt

Corresponding author(s): Fiona Watt (fiona.watt@embo.org)

Review Timeline:

Submission Date:	17th Apr 25
Accepted:	29th Apr 25

Editor: Holger Breithaupt

Transaction Report: This article was editorially reviewed.
